# Chemoembolization for Single Large Hepatocellular Carcinoma with Preserved Liver Function: Analysis of Factors Predicting Clinical Outcomes in a 302 Patient Cohort

**DOI:** 10.3390/life11080840

**Published:** 2021-08-17

**Authors:** Gun Ha Kim, Jin Hyoung Kim, Ju Hyun Shim, Heung-Kyu Ko, Hee Ho Chu, Ji Hoon Shin, Hyun-Ki Yoon, Gi-Young Ko, Dong Il Gwon

**Affiliations:** 1 Department of Radiology, Asan Medical Center, University of Ulsan College of Medicine, 88 Olympic-ro 43-gil, Songpa-gu, Seoul 05505, Korea; kimgh.rad@amc.seoul.kr (G.H.K.); hk.ko@amc.seoul.kr (H.-K.K.); angiochu@amc.seoul.kr (H.H.C.); jhshin@amc.seoul.kr (J.H.S.); hkyoon@amc.seoul.kr (H.-K.Y.); kogy@amc.seoul.kr (G.-Y.K.); radgwon@amc.seoul.kr (D.I.G.); 2 Department of Gastroenterology, Asan Medical Center, University of Ulsan College of Medicine, 88 Olympic-ro 43-gil, Songpa-gu, Seoul 05505, Korea; s5854@amc.seoul.kr

**Keywords:** hepatocellular carcinoma, transarterial chemoembolization, risk factors, survival

## Abstract

The purpose of this study was to define the role of transcatheter arterial chemoembolization (TACE) in patients with a single large hepatocellular carcinoma (HCC) and define the patient groups benefiting from TACE. Treatment-naïve patients with preserved liver function who received TACE as the first-line treatment for single large (>5 cm) HCC without macrovascular invasion and extrahepatic metastasis between 2007 and 2019 were retrospectively analyzed. Overall survival, progression-free survival, radiologic tumor response, complications, and predictors of survival were analyzed using multivariate analysis, and then a pretreatment risk-prediction model was created using the four predictive factors of tumor size, tumor type, ALBI grade, and ECOG performance status. Patients with scores of 0 (n = 54), 1–2 (n = 170), and 3–6 (n = 78) according to the model were classified as low-, intermediate-, and high-risk, respectively. The corresponding median OS values were 141, 55, and 28 months, respectively. The percentage of major complications increased as tumor size increased (4–21%). Asymptomatic, nodular HCC patients with a tumor size of 5–7 cm and ALBI grade 1 benefited the most from TACE. By contrast, the value of TACE in the treatment of single huge HCC (>10 cm) with high complication rates remains unclear.

## 1. Introduction

According to the updated Barcelona Clinic Liver Cancer (BCLC) staging system [1], single large (>5 cm) hepatocellular carcinoma (HCC) is classified as early-stage disease (BCLC A) because of higher survival when treated with surgical resection in comparison with alternative treatments [2,3]. However, surgical resection is not always suitable for single large HCC, which is not infrequently considered unresectable because of the tumor size, location, patient age, underlying liver cirrhosis, or other comorbidities [4,5]. In addition, major liver resection is typically required to treat large HCC, and some patients are reluctant to receive aggressive surgical resection.

Transcatheter arterial chemoembolization (TACE) is a treatment that has been widely used for many years to delay tumor progression in patients with unresectable HCC [6]. With the development of TACE devices and techniques, patient survival has gradually increased, and TACE-related mortality has decreased year by year [6]. Asymptomatic patients with multinodular HCCs and those with unresectable single large HCCs with preserved liver function are considered to be “recommended” or “ideal” TACE candidates [4,7].

A previous propensity score analysis (59 pairs) [8] showed that TACE had comparable survival outcomes to surgical resection when used for the treatment of single large HCC (median overall survival (OS); 76.4 vs. 60.7 months in surgical resection and TACE groups, respectively; *p* = 0.293), although the study was limited by the small sample size and retrospective nonrandomized study design. Currently, the role of TACE in the treatment of single large HCC remains poorly defined, as it has not been well studied in this regard. TACE may not always be effective in the treatment of single large HCCs, and it is, therefore, important to define the role of TACE and identify those patient groups who will benefit from TACE for the treatment of single large HCC.

In the present study, we evaluated clinical outcomes and factors predicting survival after TACE in a large cohort of 302 patients with single large HCC and preserved liver function.

## 2. Materials and Methods

### 2.1. Study Patients

Treatment-naïve patients who underwent TACE as the first-line treatment for single large (>5 cm) HCC without macrovascular invasion (MVI) and extrahepatic metastasis (EHM) between January 2007 and December 2019, were included in this analysis. Included patients received TACE as the initial treatment because they initially had unresectable HCC because of insufficient future remnant liver volume, tumor location, portal hypertension, or extrahepatic comorbidities, or because they refused surgery due to old age or personal preference. Patients were excluded if they had decompensated liver function with a Child–Pugh score ≥ 8, if they were lost to follow-up during the follow-up period, or if they had a previous or current malignancy except for HCC. The study design was approved by our institutional review board, and the requirement for patient consent was waived due to the retrospective nature of the study.

### 2.2. Transcatheter Arterial Chemoembolization

Detailed explanations of the TACE procedure have been presented in previous articles [9], and we, therefore, provide only a summary here. TACE was performed by one of seven interventional radiologists with at least 10 years of experience. TACE with lipiodol (Guerbet, Roissy, France) was performed using a cisplatin dose of 2 mg/kg. Using a 1.7–2.4-F microcatheter (Progreat Lambda, Terumo, Tokyo, Japan; Renegade, Boston Scientific, Cork, Ireland; Carnelian, Tokai Medical Products, Aichi, Japan), an emulsion of lipiodol (maximum dose, 20 mL) and cisplatin in a 1:1 ratio was infused into the feeding artery. This was then followed by Gelfoam slurry (Upjohn, Kalamazoo, MI, USA) embolization until sufficient arterial flow stasis to the segmental artery level was achieved. All HCCs were embolized in a single TACE session. After TACE, patients were observed overnight for management of possible postembolization syndrome or other complications. Patients were discharged when they had no discomfort or after resolution of complications. Initial follow-up examinations (laboratory tests and CT) were made 1 month after TACE. Thereafter, subsequent follow-up examinations (laboratory tests, and CT or MRI) were repeated every 2–3 months. Repeat TACE was performed when residual tumor, tumor growth, or new tumors were detected on follow-up CT or MRI scans until the patients’ underlying hepatic function or general condition could no longer tolerate TACE.

### 2.3. Study End Point

The primary study endpoints were patient OS and the identification of pretreatment factors for predicting OS following TACE in patients with single large HCCs. OS was defined as the interval between the first TACE procedure and either death or the last follow-up. Patients who were alive at the end of the study (March 2021) were censored for survival rate calculations. The following pretreatment factors for estimating OS were evaluated: patient age, sex, tumor size (≤7 cm, 7–10 cm, >10 cm), morphological tumor type (nodular, infiltrative), extent of tumor (unilobar, bilobar involvement), presence or absence of portal hypertension, serum alpha-fetoprotein (AFP) level (<400 ng/mL, ≥400 ng/mL) [10], serum bilirubin level, serum albumin level (>3.5 g/dL, ≤3.5 g/dL) [11], albumin–bilirubin (ALBI) grade [12], and Eastern Cooperative Oncology Group (ECOG) performance status. Portal hypertension was diagnosed according to one or more of the following: esophageal varix, noticeable portosystemic collaterals, ascites, and splenomegaly with thrombocytopenia (a platelet count <100,000/mm^3^) [13].

Secondary study endpoints were radiologic tumor response, progression-free survival (PFS), and complications following TACE. Tumor response was assessed using the mRECIST criteria, which are divided into four response categories: complete response (CR), partial response (PR), stable disease (SD), and progressive disease (PD) [14]. The initial response (at 1 month following TACE) and the best overall response (best tumor response over the whole study between initial treatment and the last tumor assessment) were evaluated [15]. Patients with a CR or PR were classified as radiologic tumor responders, and patients with SD or PD as non-responders. PFS was defined as the time elapsed between initial TACE and tumor progression (on the basis of the mRECIST guidelines) or death from any cause [16].

Using the reporting standards of the Society of Interventional Radiology (SIR) [17], major complications were defined as those necessitating additional treatment, including a hospital stay beyond the normal postoperative course, increased level of care, substantial morbidity, or death (SIR classifications C–E). All other complications were considered as minor (SIR classifications A and B). Post-embolization syndrome, including transient fever, abdominal pain, nausea, and/or vomiting, was not considered morbidity. However, if a fever persisted for more than 7 days in spite of antibiotic treatment, it was regarded as a major complication. Mortality was defined as death within 30 days from the time of TACE.

### 2.4. Statistical Analysis

A multivariate Cox-proportional hazards model using the backward elimination method was used to find pretreatment factors predicting post-TACE OS amongst those variables showing *p* < 0.05 in a univariate analysis. Risk points were then assigned to the variables according to their β regression coefficients, and a pretreatment risk-prediction model was created [18]. Three prognostic categories were identified according to changes in the risk estimate for each point increase in the score. Cumulative survival curves were created according to the Kaplan–Meier method and compared using a log-rank test. The three subgroups (according to tumor size (≤7 cm, 7–10 cm, >10 cm)) were compared using analysis of variance for continuous data and the χ^2^ test for categorical data [19]. Statistical analyses were performed using SPSS (version 21.0, SPSS, Chicago, IL, USA), and two-sided *p*-values of <0.05 were considered statistically significant.

## 3. Results

### 3.1. Patient Characteristics

**Figure 1:**
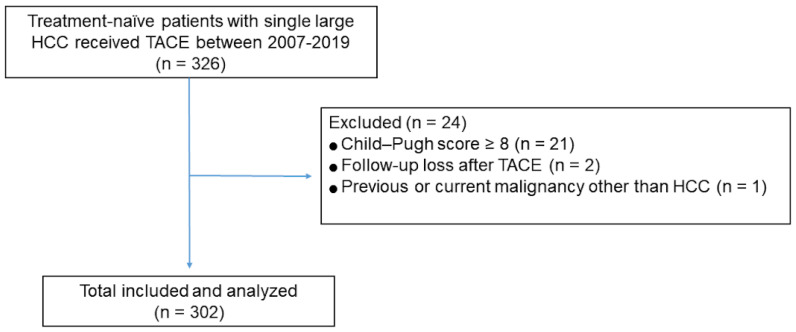
Flow diagram of the study population.

Of the 326 consecutive patients identified as having received TACE as first-line treatment for single large HCC during the study period, 302 were included in this study (Figure 1). The mean patient age was 63.1 years. The majority of study patients were men (84%), were positive for hepatitis B virus (69%), and had a nodular tumor type (96%), unilobar tumor involvement (94%), and no tumor-related symptoms (ECOG performance status of 0, 84%). A quarter (25%) of the study patients had portal hypertension. Rates of tumor-related symptoms (ECOG performance status of 1) and serum AFP ≥ 400 ng/mL were higher in tumors of larger size. The rate of bilobar tumor involvement was significantly higher in patients with huge (>10 cm) HCC than in patients with HCC of 5–7 cm or 7–10 cm, whereas the rate of portal hypertension was significantly lower in the patients with huge HCC (Table 1).

### 3.2. Model for Prediction of Overall Survival

The median number of TACE sessions per patient was 3 (range, 1–22 sessions). Two hundred and twenty patients (73%, 220/302) received repeat TACE to treat a residual tumor, tumor growth, or new tumors. Seventeen patients (6%, 17/302) underwent surgical resection (n = 9) or liver transplantation (n = 8) owing to significant size reduction after repeat TACE. During the follow-up period (median, 33 months (interquartile range, 17–58 months)), 163 (54%) patients died and 139 (46%) remained alive. The median post-TACE OS of the 302 patients was 48 months (95% confidence interval (CI), 36–60 months). The OS rates of the whole cohort at 1, 3, 5, and 10 years were 84%, 58%, 45%, and 35%, respectively.

**Figure 2:**
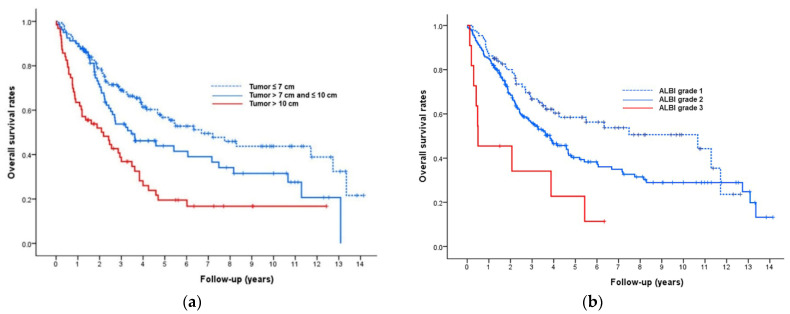

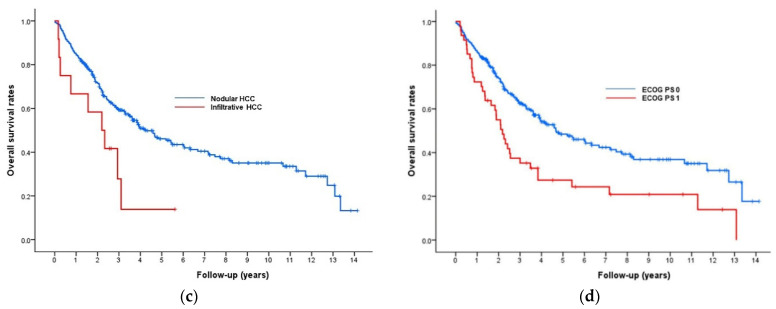
Kaplan–Meier analysis of overall survival (OS) according to tumor size, ALBI grade, tumor type, and ECOG performance status. (**a**) Kaplan–Meier curves showing OS rates according to tumor size. The median OS period was 80 months for tumors ≤ 7 cm, 42 months for tumors 7–10 cm, and 26 months for tumors > 10 cm (*p* < 0.001). (**b**) Kaplan–Meier curves showing OS rates according to ALBI grade. The median OS period was 128 months for patients with ALBI grade 1, 46 months for patients with ALBI grade 2, and 6 months for patients with ALBI grade 3 (*p* = 0.001). (**c**) Kaplan–Meier curves showing OS rates according to tumor type. The median OS period was 51 months for patients with nodular HCC and 26 months for patients with infiltrative HCC (*p* = 0.017). (**d**) Kaplan–Meier curves showing OS rates according to ECOG performance status. The median OS period was 56 months for patients with ECOG 0 and 26 months for patients with ECOG 1 (*p* < 0.001).

Multivariate Cox regression analyses showed that tumor size (adjusted hazard ratio (HR), 1.43 for tumor > 7–10 cm, 2.16 for tumor > 10 cm; *p* = 0.001), tumor type (adjusted HR, 2.21 for infiltrative tumor type; *p* = 0.030), ALBI grade (adjusted HR, 1.30 for ALBI grade 2, 3.41 for ALBI grade 3; *p* = 0.005), and ECOG performance status (adjusted HR, 1.51 for ECOG 1; *p* = 0.045) were significantly associated with the post-TACE OS rate (Table 2). Kaplan–Meier curves determined with these four factors are shown in Figure 2.

**Figure 3:**
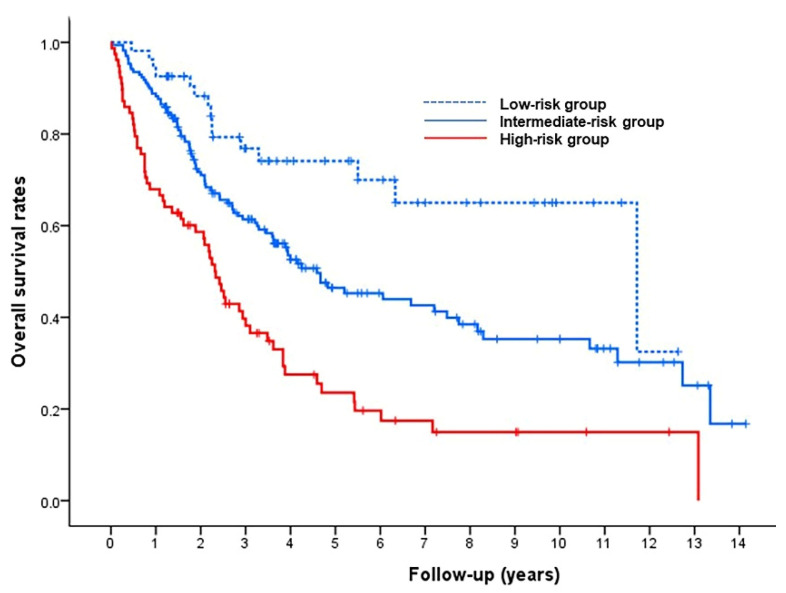
Kaplan–Meier curves stratified according to low-, intermediate-, and high-risk groups.

A pretreatment risk-prediction model was constructed using the four predictive factors identified in the multivariable Cox analysis. The β regression coefficients of the four factors and their risk points are shown in Table 2. The risk scores for all patients were estimated as the sum of these corresponding risk points, and patients with scores of 0 (n = 54), 1–2 (n = 170), and 3–6 (n = 78) were categorized into low-, intermediate-, and high-risk groups, respectively. The median OS values in the low-, intermediate-, and high-risk groups were 141 months (95% CI, 50–232 months), 55 months (95% CI, 36–74 months), and 28 months (95% CI, 23–33 months), respectively (Figure 3). OS rates were shorter in those with higher risk scores, differing significantly between the low- and intermediate-risk groups (*p* = 0.007), and between the intermediate- and high-risk groups (*p* < 0.001).

### 3.3. Radiologic Tumor Response, Progression-Free Survival, and Major Complications

**Figure 4:**
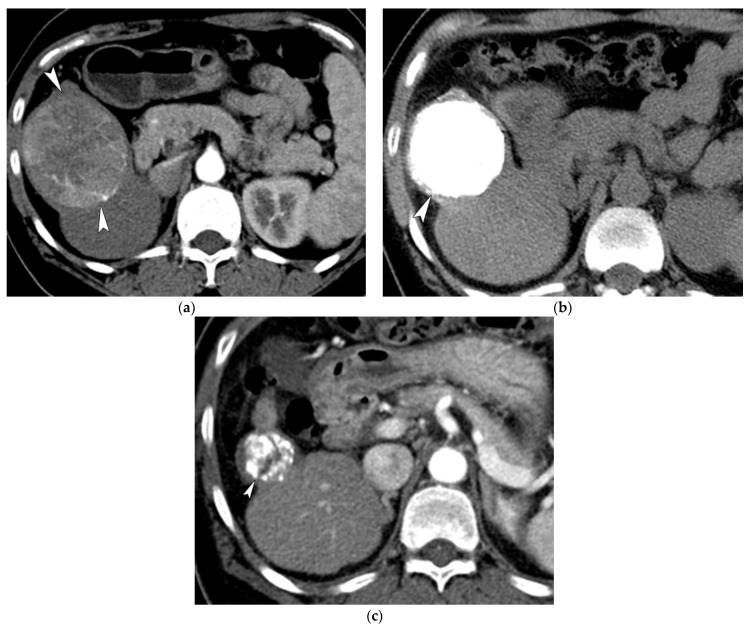
Images of a 47-year-old female patient with HCC. (**a**) Contrast-enhanced axial CT image in the arterial phase shows a large enhancing mass (7 cm in maximal diameter; arrowheads). (**b**) Non-enhanced axial CT image obtained 1 month after the initial TACE shows the presence of compact lipiodol uptake in the tumor and a small decrease in tumor size (5 cm; arrowhead). (**c**) CT at 5 years after initial TACE shows a further decrease in tumor size without recurrence (3 cm; arrowhead).

**Figure 5:**
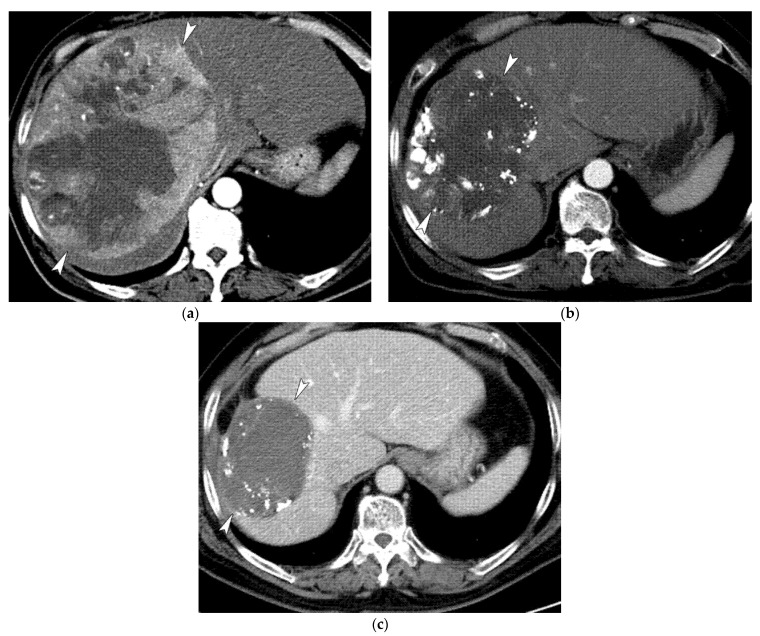
Images of a 64-year-old male patient with HCC. (**a**) Contrast-enhanced axial CT image in the arterial phase shows a huge enhancing mass (19 cm in maximal diameter; arrowheads). (**b**) CT at 1 year after five sessions of TACE shows necrotic change with lipiodol uptake in the tumor and a decrease in tumor size (13 cm; arrowheads). (**c**) CT at 5 years after eight sessions of TACE shows a further decrease in tumor size without recurrence (10 cm; arrowheads).

Assessment of tumor response at 1 month was not possible in 2 (0.7%) of the 302 patients due to mortality, and these two patients were regarded as PD. At 1-month post-TACE, 141 patients (47%) achieved a CR (Figure 4), 98 (32%) achieved a PR, 38 (13%) showed SD, and 25 (8%) showed PD. The proportion of responders (CR or PR) at initial response was 79%. During the TACE series, the best overall response achieved consisted of CR in 220 patients (73%; Figure 5), PR in 40 patients (13%), SD in 17 patients (6%), and PD in 25 patients (8%). The proportion of responders over the entire TACE series was 86%. The initial and best overall response rates according to tumor size are summarized in Table 3. The initial and best overall response rates were significantly lower in patients with a tumor size > 10 cm.

**Figure 6:**
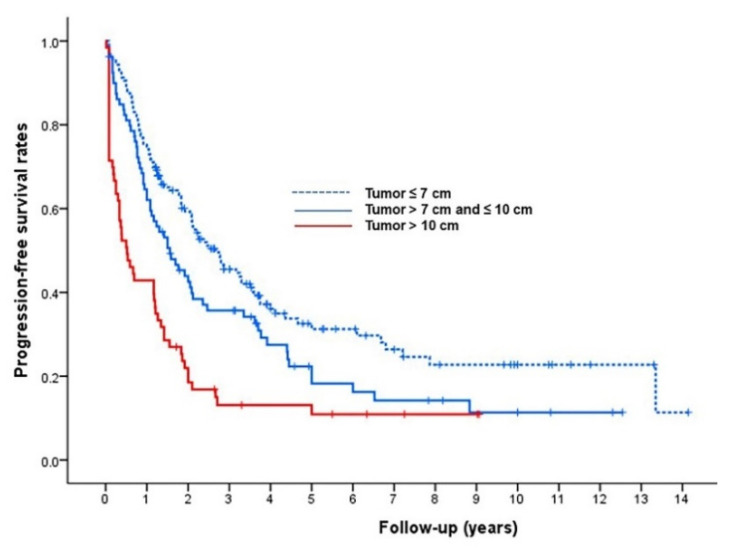
Kaplan–Meier analysis of progression-free survival (PFS) according to tumor size. The median PFS period was 32 months for tumors ≤ 7 cm, 19 months for tumors 7–10 cm, and 6 months for tumors > 10 cm (*p* < 0.001).

During follow-up, 218 patients (72%, 218/302) died or experienced progression of HCC. The median post-TACE PFS of the 302 patients was 22 months (95% CI, 18–26 months). The Kaplan–Meier curves determined with tumor size are shown in Figure 6.

After TACE, 29 of the 302 patients (10%) experienced major complications (Table 4). The major complication rates were 4% (6/159) in patients with a tumor ≤ 7 cm, 13% (10/80) in patients with a tumor 7–10 cm, and 21% (13/63) in patients with a tumor > 10 cm (*p* < 0.001). Two patients died of septic shock within 30 days after TACE; thus, the mortality rate was 0.7%.

## 4. Discussion

To the best of our knowledge, this is the largest study evaluating clinical outcomes of TACE as the first-line treatment for treatment-naïve patients with single large HCCs. Due to the large sample size (n = 302), a pretreatment prediction model could be built in this study. In our study of TACE for the treatment of single large (>5 cm) HCC in patients with preserved liver function, the median OS and OS rates of the whole cohort at 1-, 3-, and 5-years were 48 months and 84%, 58%, and 45%, respectively. In the multivariable Cox regression analysis, tumor size, tumor type, ALBI grade, and ECOG performance status were statistically significant predictors of OS. We created a pretreatment prediction model using these four factors and identified three risk groups according to risk score: low-, intermediate-, and high-risk groups. The corresponding median OS times were 141, 55, and 28 months, respectively. Our study is different from others since previous studies investigating TACE for single large HCC only included huge HCCs (>10 cm) [20,21] or performed subgroup comparison of single large HCCs with heterogenous BCLC A or B HCCs [22]. Furthermore, previous risk prediction models were generated for inhomogeneous groups [10,23,24] or single small HCC (≤3 cm) [25].

Our results indicate that asymptomatic, nodular HCC patients with a tumor size of 5–7 cm and ALBI grade 1 received the most benefit from TACE treatment (median OS: 141 months). A previous propensity score analysis found that TACE had similar OS outcomes to surgical resection in patients with a tumor size of 5–7 cm (HR: 0.70; 95% CI: 0.38–1.26; *p* = 0.230) [8]. Thus, we believe that TACE can be considered as an alternative treatment option in these patients when they have a nonresectable condition or prefer nonsurgical treatment.

In line with EASL guidelines [26] that classify a huge tumor size (>10 cm) as a relative contraindication for TACE, we found that the median OS of patients with a tumor > 10 cm in this study was only 26 months and that they had a relatively high major complication rate (21%). Four previous studies used propensity score matching to investigate the efficacy of surgery versus TACE for huge (>10 cm) HCC [27,28,29,30]. All four studies demonstrated that surgery was associated with longer median OS than TACE for patients with solitary huge HCC (Bogdanovic et al. [29]: 20 pairs, 19 vs. 13 months, respectively, *p* = 0.023; Zhu et al. [27]: 61 pairs, 37 vs. 19 months, *p* = 0.039; Min et al. [28]: 76 pairs, 38 vs. 10 months, *p* < 0.001; and Wei et al. [30]: 37 pairs, 36 vs. 12 months, *p* = 0.010). Indeed, a long-term study including 471 patients showed 1-, 3-, 5-, and 10-year OS rates of 69%, 47%, 36%, and 19%, respectively, for resection of huge HCC [31]. Thus, surgery should be considered as the initial treatment option whenever possible, even in patients with huge HCC. In addition, a multicenter propensity matching analysis (84 pairs) [32] showed that patients who underwent preoperative TACE for liver resection of huge HCC had better median OS than those who did not (33 vs. 18 months, *p* = 0.023). Furthermore, in patients with huge HCC who underwent liver resection, those who also underwent postoperative adjuvant TACE showed better survival outcomes than those who did not (69 pairs; OS rates of 43% vs. 25% at 5 years, *p* = 0.004) [33]. Therefore, for huge HCC, TACE can also be performed as a preoperative or postoperative adjunctive therapy rather than the main therapeutic strategy.

However, only a small number of patients with huge HCC are candidates for surgical resection, and other alternative salvage treatment options should be considered for patients who are initially excluded from surgery. A previous retrospective study [34] suggested that TACE combined with radiotherapy could be a better alternative option than TACE alone for the treatment of unresectable huge HCC; the median OS of TACE plus radiotherapy was significantly better than that of TACE alone (15 vs. 8 months, *p* = 0.04) in patients with huge HCC (BCLC B or C stage).

Many investigators have demonstrated the limitations of the current BCLC staging system for single large HCC (>5 cm) [35,36,37,38]. The survival outcomes of patients with single large HCC are significantly worse than those of patients with smaller HCC and are rather similar to those of BCLC B patients [3,39,40,41,42,43]. In our study, we further subdivided patients with single large (>5 cm) HCCs into three subgroups according to tumor size. We found a median OS of 80 months in patients with a tumor ≤ 7 cm, 42 months in patients with a tumor 7–10 cm, and 26 months in patients with a tumor > 10 cm. Thus, we believe that a single HCC ≤ 7 cm can be staged as BCLC A. However, the median OS of single large HCCs of 7–10 cm or > 10 cm was within the range of that of intermediate-stage HCC (median OS, 14–45 months) [1], and these can thus be staged as BCLC B.

With respect to ECOG performance status, patients are classified as advanced stage (BCLC C) when they have tumor-related symptoms, irrespective of MVI and EHM. Multiple studies have reported that an ECOG performance status of 1 alone should not be considered sufficient to allocate patients to advanced-stage disease [38,44,45], which would impede any potential therapy for HCC. The median OS of patients with ECOG performance status 1 in our study was 26 months, which was beyond the range of that of advanced-stage HCC (median OS, 6–14 months) [1]. Thus, symptomatic patients with a single large HCC without MVI and EHM could be staged as intermediate stage.

The main limitation of this study is its retrospective design, which makes potential biases unavoidable. However, we tried to minimize bias by using a relatively large population (302 consecutive patients). This study also examined patients recruited from a single tertiary hospital, and external validation of the findings is therefore needed.

In conclusion, although our analysis did not focus on a comparison of TACE with resection or other curative treatments, our results suggest that asymptomatic, nodular HCC patients with a tumor size of 5–7 cm and ALBI grade 1 benefit the most from TACE in the treatment of single large (>5 cm) HCC. By contrast, the use of TACE for the treatment of single huge HCC (>10 cm) remains questionable, with high complication rates being found, and further study is required to find a better option for the treatment of single huge HCC (>10 cm).

## Figures and Tables

**Table 1:** Study patient demographics.

	All Study Patients	Tumor Size ≤ 7 cm	Tumor Size 7–10 cm	Tumor Size > 10 cm	*p*-Value
	(n = 302)	(n = 159)	(n = 80)	(n = 63)	
Age, mean ± SD, years	63 ± 112	62 ± 11	64 ± 11	64 ± 13	0.400
Gender (%)					0.950
Male	253 (84)	134 (84)	67 (84)	52 (83)	
Female	49 (16)	25 (16)	13 (16)	11 (17)	
Etiology (%)					0.730
HBV	208 (69)	110 (69)	57 (71)	41 (65)	
Others	94 (31)	49 (31)	23 (29)	22 (35)	
Tumor type					0.737
Nodular	290 (96)	154 (97)	76 (95)	60 (95)	
Infiltrative	12 (4)	5 (3)	4 (5)	3 (5)	
Tumor involvement (%)					<0.001
Unilobar	284 (94)	156 (98)	79 (99)	49 (78)	
Bilobar	18 (6)	3 (2)	1 (1)	14 (22)	
Albumin (g/dL, mean ± SD)	3.6 ± 0.5	3.7 ± 0.5	3.6 ± 0.5	3.5 ± 0.5	0.052
Bilirubin (mg/dL, mean ± SD)	0.9 ± 0.5	0.9 ± 0.5	0.9 ± 0.5	0.9 ± 0.4	0.690
ALBI grade					0.283
1	87 (29)	53 (33)	18 (22)	16 (26)	
2	204 (67)	102 (64)	59 (74)	43 (68)	
3	11 (4)	4 (3)	3 (4)	4 (6)	
AFP ≥ 400 ng/mL (%)	86 (28)	33 (21)	23 (29)	30 (48)	<0.001
Presence of portal hypertension					0.005
Yes	77 (25)	42 (26)	28 (35)	7 (11)	
No	225 (75)	117 (74)	52 (65)	56 (89)	
ECOG PS					<0.001
0	255 (84)	148 (93)	69 (86)	38 (60)	
1	47 (16)	11 (7)	11 (14)	25 (40)	

SD, standard deviation; HBV, hepatitis B virus; AFP, alpha-fetoprotein; ALBI, albumin–bilirubin; ECOG PS, Eastern Cooperative Oncology Group performance status.

**Table 2:** Univariable and multivariable analysis of factors associated with overall survival after TACE for single large HCC.

	Univariable Regression Analysis				Multivariable Regression Analysis					
Variable	HR	95% CI		*p*-Value	HR	95% CI		*p*-Value	Beta Coefficients	Risk Point
Tumor size				<0.001				0.001		
≤7 cm	1				1					
7–10 cm	1.55	1.07	2.24		1.43	0.98	2.09		0.36	1
>10 cm	2.55	1.75	3.71		2.16	1.44	3.25		0.77	2
Portal hypertension	1.24	0.89	1.74	0.210						
Infiltrative tumor type	2.41	1.13	5.17	0.020	2.21	1.10	4.41	0.030	0.79	2
Bilobar involvement	2.24	1.30	3.89	0.004	1.45	0.77	2.71	0.250		
ECOG PS 1	1.96	1.36	2.83	<0.001	1.51	1.01	2.27	0.045	0.41	1
AFP ≥ 400 ng/mL	1.56	1.13	2.16	0.007	1.20	0.85	1.71	0.310		
Albumin ≤ 3.5 g/dL	1.61	1.18	2.19	0.002	1.20	0.82	1.75	0.350		
Bilirubin (mg/dL)	1.17	0.85	1.60	0.330						
ALBI grade				0.001				0.005		
1	1				1					
2	1.49	1.04	2.16		1.30	0.90	1.89		0.26	1
3	3.76	1.82	7.80		3.41	1.63	7.13		1.22	3
Age (years)	1.01	0.99	1.03	0.080						
Male sex	0.79	0.52	1.19	0.250						
HBV etiology	0.85	0.61	1.19	0.350						

HR, hazard ratio; CI, confidence interval; AFP, alpha-fetoprotein; ECOG PS, Eastern Cooperative Oncology Group performance status; ALBI, albumin–bilirubin; HBV, hepatitis B virus.

**Table 3:** Initial and best overall responses according to tumor size.

		All Study Patients	Tumor Size ≤ 7 cm	Tumor Size 7–10 cm	Tumor Size > 10 cm	*p*-Value
		(n = 302)	(n = 159)	(n = 80)	(n = 63)	
Initial response (%)	CR or PR	239 (79)	146 (92)	70 (88)	23 (37)	< 0.001
	SD or PD	63 (21)	13 (8)	10 (12)	40 (63)	
Best overall response (%)	CR or PR	260 (86)	150 (94)	72 (90)	38 (60)	< 0.001
	SD or PD	42 (14)	9 (6)	8 (10)	25 (40)	

CR, complete response; PR, partial response; SD, stable disease; PD, progressive disease.

**Table 4:** Major complications.

Major Complications	Number
Persistent fever > 7 days	8
Hepatic abscess	5
Sepsis	4
TACE-related cholecystitis	3
Hepatic failure	2
Tumor lysis syndrome	2
Biloma	2
Contrast agent-associated ARF	1
Spontaneous bacterial peritonitis	1
Tumor rupture	1

TACE, transarterial chemoembolization; ARF, acute renal failure.

## Data Availability

Not applicable.

## References

[1] European Association For The Study Of The Liver, European Organisation For Research And Treatment Of Cancer (2012). EASL-EORTC clinical practice guidelines: Management of hepatocellular carcinoma. J. Hepatol..

[2] Bruix J., Gores G.J., Mazzaferro V. (2014). Hepatocellular carcinoma: Clinical frontiers and perspectives. Gut.

[3] Vitale A., Burra P., Frigo A.C., Trevisani F., Farinati F., Spolverato G., Volk M., Giannini E.G., Ciccarese F., Piscaglia F. (2015). Survival benefit of liver resection for patients with hepatocellular carcinoma across different Barcelona Clinic Liver Cancer stages: A multicentre study. J. Hepatol..

[4] Wang Q., Xia D., Bai W., Wang E., Sun J., Huang M., Mu W., Yin G., Li H., Zhao H. (2019). Development of a prognostic score for recommended TACE candidates with hepatocellular carcinoma: A multicentre observational study. J. Hepatol..

[5] Centonze L., Di Sandro S., Lauterio A., De Carlis R., Frassoni S., Rampoldi A., Tuscano B., Bagnardi V., Vanzulli A., De Carlis L. (2021). Surgical Resection vs. Percutaneous Ablation for Single Hepatocellular Carcinoma: Exploring the Impact of Li-RADS Classification on Oncological Outcomes. Cancers.

[6] Hu H.T., Kim J.H., Lee L.S., Kim K.A., Ko G.Y., Yoon H.K., Sung K.B., Gwon D.I., Shin J.H., Song H.Y. (2011). Chemoembolization for hepatocellular carcinoma: Multivariate analysis of predicting factors for tumor response and survival in a 362-patient cohort. J. Vasc. Interv. Radiol..

[7] Iezzi R., Bilhim T., Crocetti L., Peynircioglu B., Goldberg S., Bilbao J.I., Sami A., Akhan O., Scalise P., Giuliante F. (2020). “Primum Non Nocere” in Interventional Oncology for Liver Cancer: How to Reduce the Risk for Complications?. Life.

[8] Lee Y.B., Lee D.H., Cho Y., Yu S.J., Lee J.H., Yoon J.H., Lee H.S., Kim H.C., Yi N.J., Lee K.W. (2015). Comparison of transarterial chemoembolization and hepatic resection for large solitary hepatocellular carcinoma: A propensity score analysis. J. Vasc. Interv. Radiol..

[9] Gaba R.C., Lokken R.P., Hickey R.M., Lipnik A.J., Lewandowski R.J., Salem R., Brown D.B., Walker T.G., Silberzweig J.E., Baerlocher M.O. (2017). Quality Improvement Guidelines for Transarterial Chemoembolization and Embolization of Hepatic Malignancy. J. Vasc. Interv. Radiol..

[10] Kim B.K., Shim J.H., Kim S.U., Park J.Y., Kim D.Y., Ahn S.H., Kim K.M., Lim Y.S., Han K.H., Lee H.C. (2016). Risk prediction for patients with hepatocellular carcinoma undergoing chemoembolization: Development of a prediction model. Liver Int..

[11] Lee I.C., Hung Y.W., Liu C.A., Lee R.C., Su C.W., Huo T.I., Li C.P., Chao Y., Lin H.C., Hou M.C. (2019). A new ALBI-based model to predict survival after transarterial chemoembolization for BCLC stage B hepatocellular carcinoma. Liver Int..

[12] Pinato D.J., Kaneko T., Saeed A., Pressiani T., Kaseb A., Wang Y., Szafron D., Jun T., Dharmapuri S., Naqash A.R. (2020). Immunotherapy in Hepatocellular Cancer Patients with Mild to Severe Liver Dysfunction: Adjunctive Role of the ALBI Grade. Cancers.

[13] Chu H.H., Kim J.H., Kim P.N., Kim S.Y., Lim Y.S., Park S.H., Ko H.K., Lee S.G. (2019). Surgical resection versus radiofrequency ablation very early-stage HCC (≤2 cm Single HCC): A propensity score analysis. Liver Int..

[14] Lencioni R., Llovet J.M. (2010). Modified RECIST (mRECIST) assessment for hepatocellular carcinoma. Semin. Liver Dis..

[15] Park C., Chu H.H., Kim J.H., Kim S.Y., Alrashidi I., Gwon D.I., Yoon H.K., Kim N. (2020). Clinical Significance of the Initial and Best Responses after Chemoembolization in the Treatment of Intermediate-Stage Hepatocellular Carcinoma with Preserved Liver Function. J. Vasc. Interv. Radiol..

[16] Saad E.D., Katz A. (2009). Progression-free survival and time to progression as primary end points in advanced breast cancer: Often used, sometimes loosely defined. Ann. Oncol..

[17] Brown D.B., Gould J.E., Gervais D.A., Goldberg S.N., Murthy R., Millward S.F., Rilling W.S., Geschwind J.F., Salem R., Vedantham S. (2009). Transcatheter therapy for hepatic malignancy: Standardization of terminology and reporting criteria. J. Vasc. Interv. Radiol..

[18] Uno H., Cai T., Tian L., Wei L.J. (2007). Evaluating Prediction Rules for t-Year Survivors With Censored Regression Models. J. Am. Stat. Assoc..

[19] Chu H.H., Kim J.H., Yoon H.K., Ko H.K., Gwon D.I., Kim P.N., Sung K.B., Ko G.Y., Kim S.Y., Park S.H. (2019). Chemoembolization Combined with Radiofrequency Ablation for Medium-Sized Hepatocellular Carcinoma: A Propensity-Score Analysis. J. Vasc. Interv. Radiol..

[20] Xue T., Le F., Chen R., Xie X., Zhang L., Ge N., Chen Y., Wang Y., Zhang B., Ye S. (2015). Transarterial chemoembolization for huge hepatocellular carcinoma with diameter over ten centimeters: A large cohort study. Med. Oncol..

[21] Miyayama S., Kikuchi Y., Yoshida M., Yamashiro M., Sugimori N., Ikeda R., Okimura K., Sakuragawa N., Ueda T., Sanada T. (2019). Outcomes of conventional transarterial chemoembolization for hepatocellular carcinoma ≥10 cm. Hepatol. Res..

[22] Fang K.C., Kao W.Y., Su C.W., Chen P.C., Lee P.C., Huang Y.H., Huo T.I., Chang C.C., Hou M.C., Lin H.C. (2018). The Prognosis of Single Large Hepatocellular Carcinoma Was Distinct from Barcelona Clinic Liver Cancer Stage A or B: The Role of Albumin-Bilirubin Grade. Liver Cancer.

[23] Han G., Berhane S., Toyoda H., Bettinger D., Elshaarawy O., Chan A.W.H., Kirstein M., Mosconi C., Hucke F., Palmer D. (2020). Prediction of Survival Among Patients Receiving Transarterial Chemoembolization for Hepatocellular Carcinoma: A Response-Based Approach. Hepatology.

[24] Nam J.Y., Choe A.R., Sinn D.H., Lee J.H., Kim H.Y., Yu S.J., Kim Y.J., Yoon J.H., Lee J.M., Chung J.W. (2020). A differential risk assessment and decision model for Transarterial chemoembolization in hepatocellular carcinoma based on hepatic function. BMC Cancer.

[25] Park C., Kim J.H., Kim P.H., Kim S.Y., Gwon D.I., Chu H.H., Park M., Hur J., Kim J.Y., Kim D.J. (2021). Imaging Predictors of Survival in Patients with Single Small Hepatocellular Carcinoma Treated with Transarterial Chemoembolization. Korean J. Radiol..

[26] Sieghart W., Hucke F., Peck-Radosavljevic M. (2015). Transarterial chemoembolization: Modalities, indication, and patient selection. J. Hepatol..

[27] Zhu S.L., Zhong J.H., Ke Y., Ma L., You X.M., Li L.Q. (2015). Efficacy of hepatic resection vs transarterial chemoembolization for solitary huge hepatocellular carcinoma. World J. Gastroenterol..

[28] Min Y.W., Lee J.H., Gwak G.Y., Paik Y.H., Lee J.H., Rhee P.L., Koh K.C., Paik S.W., Yoo B.C., Choi M.S. (2014). Long-term survival after surgical resection for huge hepatocellular carcinoma: Comparison with transarterial chemoembolization after propensity score matching. J. Gastroenterol. Hepatol..

[29] Bogdanovic A., Bulajic P., Masulovic D., Bidzic N., Zivanovic M., Galun D. (2021). Liver resection versus transarterial chemoembolization for huge hepatocellular carcinoma: A propensity score matched analysis. Sci. Rep..

[30] Wei C.Y., Chen P.C., Chau G.Y., Lee R.C., Chen P.H., Huo T.I., Huang Y.H., Su Y.H., Hou M.C., Wu J.C. (2020). Comparison of prognosis between surgical resection and transarterial chemoembolization for patients with solitary huge hepatocellular carcinoma. Ann. Transl. Med..

[31] Hwang S., Lee Y.J., Kim K.H., Ahn C.S., Moon D.B., Ha T.Y., Song G.W., Jung D.H., Lee S.G. (2015). Long-Term Outcome After Resection of Huge Hepatocellular Carcinoma ≥ 10 cm: Single-Institution Experience with 471 Patients. World J. Surg..

[32] Li C., Wang M.D., Lu L., Wu H., Yu J.J., Zhang W.G., Pawlik T.M., Zhang Y.M., Zhou Y.H., Gu W.M. (2019). Preoperative transcatheter arterial chemoembolization for surgical resection of huge hepatocellular carcinoma (≥ 10 cm): A multicenter propensity matching analysis. Hepatol. Int..

[33] Wang H., Yu H., Qian Y.W., Cao Z.Y., Wu M.C., Cong W.M. (2021). Postoperative adjuvant transcatheter arterial chemoembolization improves the prognosis of patients with huge hepatocellular carcinoma. Hepatobiliary Pancreat. Dis. Int..

[34] Kim K.H., Kim M.S., Chang J.S., Han K.H., Kim D.Y., Seong J. (2014). Therapeutic benefit of radiotherapy in huge (≥10 cm) unresectable hepatocellular carcinoma. Liver Int..

[35] Vitale A., Trevisani F., Farinati F., Cillo U. (2020). Treatment of Hepatocellular Carcinoma in the Precision Medicine Era: From Treatment Stage Migration to Therapeutic Hierarchy. Hepatology.

[36] Di Sandro S., Centonze L., Pinotti E., Lauterio A., De Carlis R., Romano F., Gianotti L., De Carlis L. (2019). Surgical and oncological outcomes of hepatic resection for BCLC-B hepatocellular carcinoma: A retrospective multicenter analysis among 474 consecutive cases. Updates Surg..

[37] Vitale A., Farinati F., Pawlik T.M., Frigo A.C., Giannini E.G., Napoli L., Ciccarese F., Rapaccini G.L., Di Marco M., Caturelli E. (2019). The concept of therapeutic hierarchy for patients with hepatocellular carcinoma: A multicenter cohort study. Liver Int..

[38] Giannini E.G., Bucci L., Garuti F., Brunacci M., Lenzi B., Valente M., Caturelli E., Cabibbo G., Piscaglia F., Virdone R. (2018). Patients with advanced hepatocellular carcinoma need a personalized management: A lesson from clinical practice. Hepatology.

[39] Pelizzaro F., Penzo B., Peserico G., Imondi A., Sartori A., Vitale A., Cillo U., Giannini E.G., Forgione A., Ludovico Rapaccini G. (2021). Monofocal hepatocellular carcinoma: How much does size matter?. Liver Int..

[40] Cho Y., Sinn D.H., Yu S.J., Gwak G.Y., Kim J.H., Yoo Y.J., Jun D.W., Kim T.Y., Lee H.Y., Cho E.J. (2016). Survival Analysis of Single Large (>5 cm) Hepatocellular Carcinoma Patients: BCLC A versus B. PLoS ONE.

[41] Liu P.H., Su C.W., Hsu C.Y., Hsia C.Y., Lee Y.H., Huang Y.H., Lee R.C., Lin H.C., Huo T.I. (2016). Solitary Large Hepatocellular Carcinoma: Staging and Treatment Strategy. PLoS ONE.

[42] Jung Y.K., Jung C.H., Seo Y.S., Kim J.H., Kim T.H., Yoo Y.J., Kang S.H., Yim S.Y., Suh S.J., An H. (2016). BCLC stage B is a better designation for single large hepatocellular carcinoma than BCLC stage A. J. Gastroenterol. Hepatol..

[43] Chapiro J., Geschwind J.F. (2014). Hepatocellular carcinoma: Have we finally found the ultimate staging system for HCC?. Nat. Rev. Gastroenterol. Hepatol..

[44] Hsu C.Y., Lee Y.H., Hsia C.Y., Huang Y.H., Su C.W., Lin H.C., Lee R.C., Chiou Y.Y., Lee F.Y., Huo T.I. (2013). Performance status in patients with hepatocellular carcinoma: Determinants, prognostic impact, and ability to improve the Barcelona Clinic Liver Cancer system. Hepatology.

[45] Golfieri R., Bargellini I., Spreafico C., Trevisani F. (2019). Patients with Barcelona Clinic Liver Cancer Stages B and C Hepatocellular Carcinoma: Time for a Subclassification. Liver Cancer.

